# The Bullets He Carried

**DOI:** 10.5811/westjem.2020.5.48216

**Published:** 2020-08-07

**Authors:** Stephen W. Hargarten

**Affiliations:** Medical College of Wisconsin, Comprehensive Injury Center, Milwaukee, Wisconsin

The Sandy Hook Elementary School mass shooting on December 14, 2012, killed 26 people including 20 young children ages six to seven. The Sandy Hook shooter fired 154 bullets in less than four minutes, or about 38 bullets per minute from a semiautomatic rifle.

When the bullet leaves a Bushmaster rifle, it travels over 2000 feet per second. This velocity gives this bullet its devastating wounding potential. As this rifle bullet penetrates a human body, the energy of the bullet tears and shreds through tissue and bone, resulting in fractures, ruptured livers, and swollen brains, leading to hemorrhage, shock, and death. As an emergency physician, I have cared for hundreds of patients injured by bullets. I have had to tell parents that their teenager has died. Even those who survive are forever maimed and suffering. As a physician, I am interested in better understanding this pathogen of gun violence: the bullet and the guns that carry them.^1^

Recently, my colleagues and I at the Medical College of Wisconsin’s Comprehensive Injury Center focused our attention on the bullet and its energy. This energy is a measure of the potential for causing wounds. Other factors play a role in wounding including the mass of the bullet and the direct tearing of tissues. But understanding the energy of a bullet and its wounding potential can help develop better treatment of the wounds.

Using the latest in high-speed video cameras, we discharged bullets through gelatin, which is commonly used to mimic human tissue. We measured the kinetic energy release of a modern, high-speed rifle bullet, and of a musket ball similar to those used in the 1780s (https://www.mcw.edu/departments/comprehensive-injury-center/research). Note the dramatic difference in speed, cavitation, wave propagation, and resultant tissue damage of the rifle bullet vs the musket ball. We found that the rifle bullet’s energy release was over nine times greater than the musket ball because of the rifle bullet’s significantly greater velocity compared to the musket ball’s velocity.

In 1789, when the Second Amendment was passed by Congress, the average number of musket balls that could be fired by a member of the militia was about two per minute. Using this number-of-bullets-released-per-minute comparison, the Sandy Hook mass shooter represented the equivalent of 19 militiamen storming the elementary school. Even worse, the energy of the rifle bullet released by the Sandy Hook mass shooter was in turn at least nine times greater per bullet than the energy released by the musket balls shot by the militia. Using this energy-release-per-minute calculation, and its accompanying wounding potential, the number of bullets and their energy fired by the Sandy Hook shooter equaled an estimated 171 militiamen storming the school. The rifle and bullet technology of 2020 far exceeds that available 230 years ago. When Congress passed the Second Amendment, they could not have anticipated that, in 2012, a single man in Connecticut would use a weapon with the killing power of an army of 171 members of the Connecticut militia.

Understanding and addressing today’s bullets, their energy, their wounding potential, and the weapons that carry them are essential elements in any comprehensive solution to gun violence. It is of critical importance that all sectors of civil society understand this energy focus when discussing policies about these bullets and the guns that carry them.

## References

[1] Hargarten SW, Lerner EB, Gorelick M (2018). Gun Violence: A Biopsychosocial Disease. West J Emerg Med.

